# Changing patterns of opioid initiation for pain management in Ontario, Canada: A population-based cross-sectional study

**DOI:** 10.1371/journal.pone.0278508

**Published:** 2022-12-08

**Authors:** Tara Gomes, Siyu Men, Tonya J. Campbell, Mina Tadrous, Muhammad M. Mamdani, J. Michael Paterson, David N. Juurlink

**Affiliations:** 1 Unity Health Toronto, Toronto, Ontario, Canada; 2 ICES, Toronto, Ontario, Canada; 3 Leslie Dan Faculty of Pharmacy, University of Toronto, Toronto, Ontario, Canada; 4 Institute of Health Policy, Management and Evaluation, University of Toronto, Toronto, Ontario, Canada; 5 Women’s College Hospital, Toronto, Ontario, Canada; 6 Department of Family Medicine, McMaster University, Hamilton, Ontario, Canada; Stanford University School of Medicine, UNITED STATES

## Abstract

**Introduction:**

The recent publication of a national guideline and quality standards in Canada have provided clinicians with new, evidence-based recommendations on safe, appropriate opioid use. We sought to characterize how well opioid initiation practices aligned with these recommendations before and following their release.

**Methods:**

We conducted a population-based study among people initiating opioids prior to the release of national guidelines (April 2015—March 2016; fiscal year [FY] 2015) and in the most recent year available (January—December 2019) in Ontario, Canada. We used linked administrative claims data to ascertain the apparent indication for opioid therapy, and characterized the initial daily dose (milligrams morphine or equivalent; MME) and prescription duration for each indication.

**Results:**

In FY2015, 653,885 individuals commenced opioids, compared to 571,652 in 2019. Over time, there were small overall reductions in the prevalence of initial daily doses exceeding 50MME (23.9% vs. 20.1%) and durations exceeding 7 days (17.4% vs. 14.8%); but the magnitude of the reductions varied widely by indication. The prevalence of high dose (>50MME) initial prescriptions reduced significantly across all indications, with the exception of dentist-prescribed opioids (13.6% vs. 12.1% above 50MME). In contrast, there was little change in initial durations exceeding 7 days across most indications, with the exception of some surgical indications (e.g. common excision; 9.3% vs. 6.2%) and among those in palliative care (35.2% vs. 29.2%).

**Conclusion:**

Despite some modest reductions in initiation of high dose and long duration prescription opioids between 2015 and 2019, clinical practice is highly variable, with opioid prescribing practices influenced by clinical indication. These findings may help identify medical specialties well-suited to targeted interventions to promote safer opioid prescribing.

## Introduction

Opioid-related harm continues to rise across North America, with nearly 50,000 people in the United States (U.S.) and nearly 5,000 Canadians dying of fatal opioid overdoses in 2018 [1,2]. Although the unregulated opioid drug supply is currently the main cause of deaths [3], evidence suggests that decades of liberal prescribing of opioids for the treatment of acute and chronic pain contributed to initial rises in opioid-related harm and the high prevalence of opioid use disorder across North America [4,5]. In response to emerging evidence about the safety of prescription opioids and concerns about unsafe prescribing, evidence-based clinical guidelines and standards were developed to support clinical decision-making at time of opioid initiation to mitigate potential harm. In the U.S., national guidelines for prescribing opioids to manage chronic pain were released in March 2016 [6] and the Canadian counterpart was published in May 2017 [7]. Additionally, quality standards related to opioid prescribing for acute [8] and chronic [9] pain for Ontario were released in March 2018.

A core recommendation of each of these documents was that initial opioid therapy should not exceed 50 milligrams of morphine equivalents (MME) per day [6,7], because higher doses are associated with increased risks of continued opioid use, opioid use disorder and opioid overdose [10–12]. In addition, guidance suggests that initial prescription durations longer than one week are rarely justified to treat acute pain [8]. These recommendations were introduced at a time when opioid prescribing was declining, but still quite liberal [13–15]. For example, in 2016, 24% of people newly initiating opioids in Ontario received daily doses above 50 MME/day and 17% received more than a 7-day supply [13].

Upon their release, the guideline and quality standards garnered considerable attention, and they have since been incorporated into medical school curricula and academic detailing for clinicians across Canada. However, little is known about how clinical practice aligned with these guidelines in the years following their release. Therefore, we sought to compare the clinical indications for opioid initiation and the characteristics of initial prescriptions before and after the release of a national guideline and quality standards in Ontario, Canada.

## Materials and methods

### Setting

We conducted two population-based retrospective cross-sectional studies of all Ontarians newly dispensed an opioid during two one-year study periods. The first cohort was accrued between April 1, 2015 and March 31, 2016, prior to the release of new guidelines and quality standards in the U.S. and Canada. The second was accrued between January 1, 2019 and December 31, 2019, the most recent year of data available. Ontario is Canada’s most populous province, with a population of 14.6 million in 2019, representing 39% of the Canadian population. The use of data in this project was authorized under section 45 of Ontario’s Personal Health Information Protection Act, which does not require review by a Research Ethics Board or patient consent.

### Data sources

We obtained data from ICES (formerly known as the Institute for Clinical Evaluative Sciences), an independent, non-profit research institute whose legal status under Ontario’s health information privacy law allows for the collection and analysis of health care and demographic data. We used the Narcotics Monitoring System (NMS) to identify all prescription opioids dispensed from retail pharmacies over both study periods (which includes all opioids with the exception of those sold over-the-counter). Data captured in the NMS includes drug identification number (DIN), prescriber identifiers, patient identifiers, dispensing date, quantity dispensed, and days supplied for all opioid prescriptions regardless of payer. We used the Canadian Institute for Health Information (CIHI) Discharge Abstract Database, Same Day Surgery Database, and National Ambulatory Care Reporting System to capture diagnoses and procedures occurring during inpatient hospitalizations, outpatient surgical procedures and emergency department visits, respectively. We identified physician services using the Ontario Health Insurance Plan (OHIP) database, and used the Ontario Cancer Registry and the Cancer Activity Level Reporting database to define prior cancer diagnoses and details on cancer treatment and palliative care. Finally, we used the OHIP Registered Persons Database to determine demographic characteristics and location of residence. These databases have high levels of completeness and are regularly used in health services research [10,13,16]. The datasets were linked using unique encoded identifiers and analyzed at ICES using SAS Enterprise Guide (version 7.1; SAS Institute Inc., Cary, NC, USA).

### Study patients

The first (ie. pre-guideline) cohort was based on a previously defined cohort of new opioid recipients used in a study examining indications for therapy and initial prescription characteristics [13]. We constructed the post-guideline cohort using an identical approach, defining new opioid recipients as those dispensed an opioid to treat pain in calendar year 2019, but who had not received a prescription for any opioid between the index date and April 1, 2016 (to align with the lookback used when creating the pre-guideline cohort). Opioids used as antitussives, antidiarrheals, opioid agonist therapy or in medical assistance in dying were excluded from both cohorts using specific drug and product identification numbers. We restricted both cohorts to people with valid Ontario health insurance to allow for data linkage, and excluded patients who were treated for an opioid overdose in an emergency department or hospital (defined by diagnosis codes T40.0-T40.4 or T40.6 from the *International Statistical Classification of Diseases and Related Health Problems*, 10th revision (ICD-10)) in the 2 years prior to the index date. We defined an individual’s index date as the date of their first receipt of a prescription opioid in the accrual period.

### Identifying the apparent clinical indication

Using a previously published stepwise hierarchical approach, we identified the clinical indication for opioid initiation among individuals in each cohort using administrative healthcare databases [13]. Because the indication for opioid therapy is not specified in the NMS, this hierarchy is based on the degree of certainty that the healthcare encounter warranted treatment with an opioid. The hierarchy first uses prescriber information to classify individuals whose index opioid was prescribed by a dentist as having an indication related to dental pain. Next, we identified individuals in receipt of palliative care services in the year prior to the index opioid prescription, followed by those with evidence of active cancer during the same timeframe, allocating them to their respective indications. Among all remaining individuals in each cohort, we captured their most proximate healthcare interaction (emergency department visit, inpatient hospitalization, physician visit or outpatient surgical procedure) on the day of opioid initiation or the 5 days preceding it, to identify all relevant diagnoses and procedures that might prompt opioid therapy.

In the subsequent hierarchical steps, we defined procedure-based indications using *Canadian Classification of Health Interventions* procedure codes on recent healthcare interactions. Among those who did not have a procedure, we defined diagnosis-based indications using ICD-10 diagnosis codes or OHIP diagnosis codes on healthcare records. All remaining individuals not classified into an indication group using the methods outlined above, as well as those with no evidence of a recent healthcare encounter, were classified into an “Unknown” group. In all, we defined 6 indication clusters and 23 specific clinical indications. Details on the hierarchy used to assign indications can be found in the Supplementary Appendix and previous publication [13].

### Prescription and patient characteristics

We defined patient demographic characteristics (age, sex, neighbourhood income quintile, rural vs urban location of residence) and initial prescription characteristics (formulation, daily dose and duration [days’ supply]) among new opioid recipients in both annual cohorts. Those with unknown geographic data had their income quintile and rurality categorized separately as missing. For each indication, we calculated the prevalence of initial daily doses exceeding 50 MME, and initial durations exceeding 7 days. If people were dispensed more than one opioid at the index date, we used the maximum days’ supply and summed the opioid doses (in MME) to calculate a daily dose. Daily doses were converted to MME using previously published methods [17]. The 50 MME threshold was used because it reflects the starting daily dose recommendations in both U.S. and Canadian guidelines [6,7]. When capturing prescription duration, we selected a threshold of seven days because evidence suggests higher risk of long-term use with prescriptions exceeding seven days [16,18,19], and it aligns with recommendations from quality standards and guidelines for acute pain [6,8].

### Statistical analysis

We summarized descriptive characteristics and the prevalence of high dose and long duration initial prescriptions using medians and interquartile ranges, and percentages as appropriate. We used standardized differences to compare characteristics between each annual cohort, using a threshold of 0.10 to indicate a meaningful difference [20].

## Results

**Fig 1:**
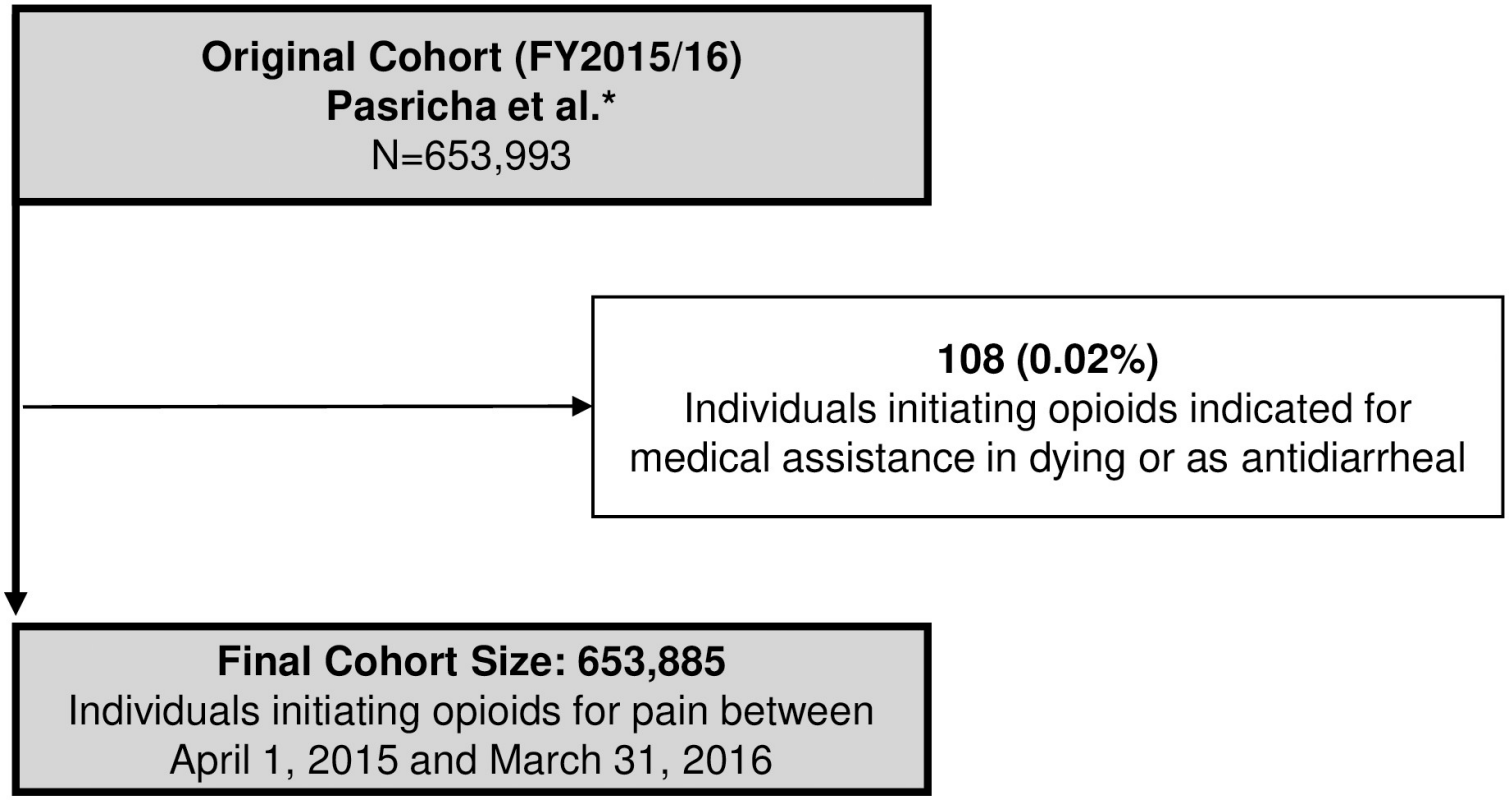
Cohort inclusion and exclusion criteria: Fiscal year 2015/16 cohort. Note that the antidiarrheal exclusion is noted here as it was not originally part of the exclusion criteria in Pasricha et al. and therefore required further exclusion for this analysis.

**Fig 2:**
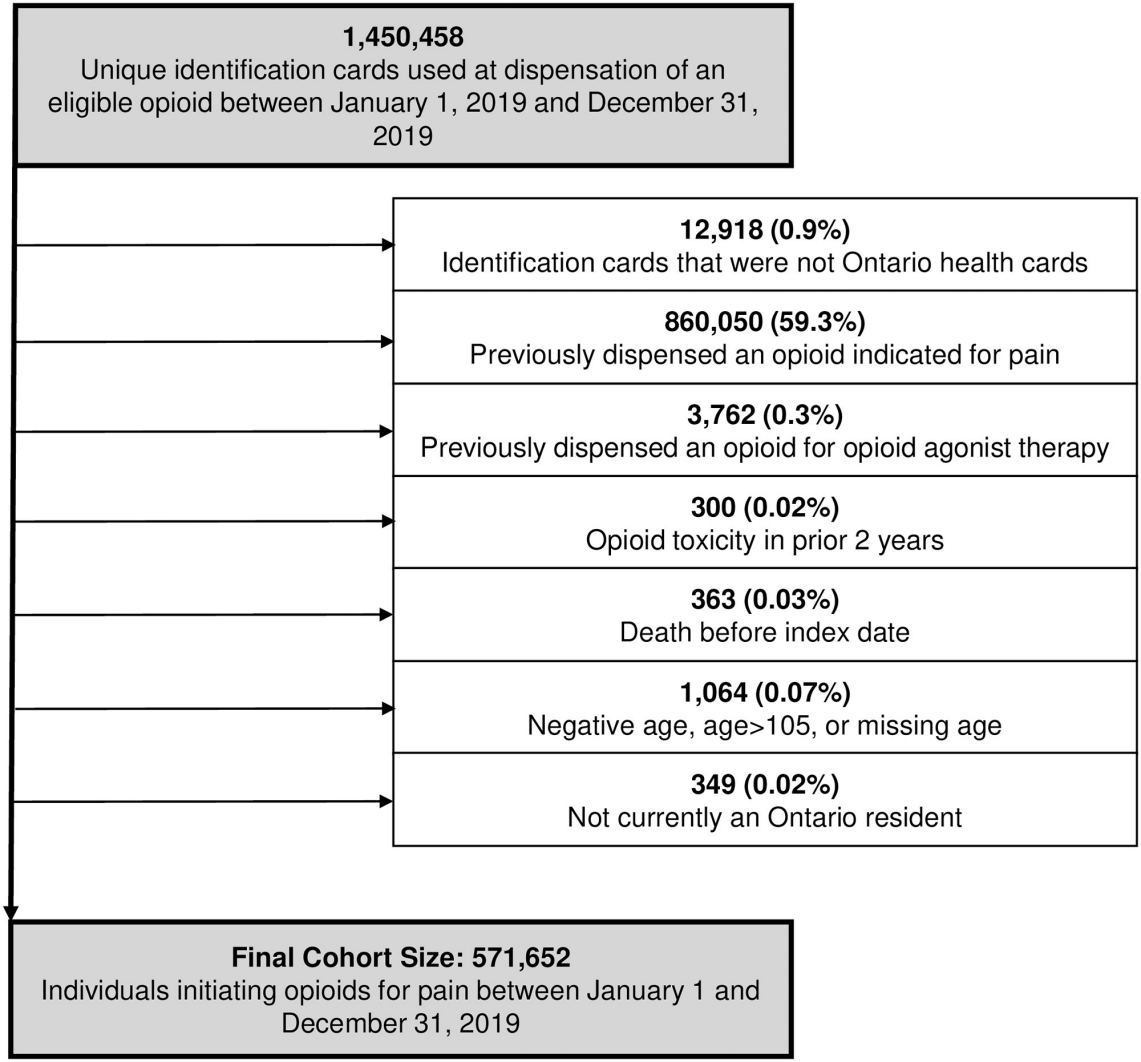
Cohort inclusion and exclusion criteria: Calendar year 2019 cohort.

A total of 653,885 and 571,652 individuals met our inclusion criteria in fiscal year (FY) 2015 and calendar year 2019, respectively (Figs 1 and 2) representing a 12.6% absolute reduction in the number of new opioid recipients. Demographic characteristics were generally similar between the cohorts, with a median age of 48 (FY2015) and 49 (2019) years, and women representing slightly more than half of new opioid recipients (51.9% vs. 52.8% in FY2015 vs. 2019; Table 1). The main difference observed across cohorts was that the median daily dose declined slightly in 2019 (34 MME vs. 30 MME; standardized difference [Std Diff] 0.16), as did the median duration (4 days vs. 3 days; Std Diff 0.21) relative to 2015. Overall, 78,468 (12.0%) individuals in FY2015 and 65,459 (11.5%) individuals in 2019 could not be linked to an indication due to either having no recent healthcare encounter identified in our data or having a healthcare record that would not normally warrant opioid initiation.

**Table 1:** Baseline characteristics of new opioid recipients in fiscal year 2015 and calendar year 2019.

Characteristic	April 1, 2015 to March 31, 2016 N = 653,885	January 1 to December 31, 2019 N = 571,652	Standardized difference
**Age (Median, IQR)**	48 (29–63)	49 (30–65)	0.06
0–17	49,450 (7.6%)	39,598 (6.9%)	0.02
18–24	70,963 (10.9%)	59,391 (10.4%)	0.02
25–44	176,896 (27.1%)	152,587 (26.7%)	0.01
45–64	205,902 (31.5%)	173,911 (30.4%)	0.02
65+	150,674 (23.0%)	146,165 (25.6%)	0.06
**Female Sex**	339,474 (51.9%)	301,687 (52.8%)	0.02
**Income quintile**			
1 (lowest)	117,216 (17.9%)	108,626 (19.0%)	0.03
2	123,416 (18.9%)	110,518 (19.3%)	0.01
3	130,235 (19.9%)	114,263 (20.0%)	0
4	142,949 (21.9%)	116,415 (20.4%)	0.04
5 (highest)	136,707 (20.9%)	120,269 (21.0%)	0
Missing	3,362 (0.5%)	1,561 (0.3%)	0.04
**Urban Location of residence**	573,016 (87.6%)	505,668 (88.5%)	0.03
Missing	363 (0.1%)	1,338 (0.2%)	0.05
**Formulation initiated**			
Immediate release only	644,654 (98.6%)	564,502 (98.7%)	0.01
Long-acting only	4,039 (0.6%)	2,030 (0.4%)	0.04
Both	5,192 (0.8%)	5,120 (0.9%)	0.01
Daily dose* **(Median, IQR)**	34 (21–45)	30 (18–45)	0.16
<20	152,483 (23.3%)	155,688 (27.2%)	0.09
20–49	334,864 (51.2%)	292,270 (51.1%)	0
50–89	126,045 (19.3%)	93,008 (16.3%)	0.08
90–199	29,126 (4.5%)	21,391 (3.7%)	0.04
> = 200	1,290 (0.2%)	643 (0.1%)	0.02
Unknown	10,077 (1.5%)	8,652 (1.5%)	0
**Days supplied (Median, IQR)**	4 (3–6)	3 (2–5)	0.21
1	18,688 (2.9%)	39,122 (6.8%)	0.19
2–3	261,755 (40.0%)	255,626 (44.7%)	0.09
4–6	219,737 (33.6%)	160,231 (28.0%)	0.12
7	40,188 (6.1%)	32,295 (5.6%)	0.02
8–13	62,130 (9.5%)	45,395 (7.9%)	0.06
14	4,884 (0.7%)	4,889 (0.9%)	0.01
15–29	27,018 (4.1%)	20,225 (3.5%)	0.03
30	16,864 (2.6%)	12,079 (2.1%)	0.03
31+	2,621 (0.4%)	1,790 (0.3%)	0.01

*in milligrams morphine or equivalent.

We observed little change in the indications for opioid initiation between FY2015 and 2019 (Table 2). In general, there were small changes in the proportion of people being treated for cancer and palliative care (increasing from 6.5% to 7.6%), trauma-related pain (decreasing from 11.2% to 9.4%), musculoskeletal pain (decreasing from 12.0% to 9.7%), and other types of pain (from 17.7% to 15.0%). The only indication where the standardized difference exceeded 0.10 was post-surgical pain (from 17.4% to 22.5%; Std Diff, 0.13). In both annual cohorts, nearly one-quarter of all new opioid treatment courses were for dental pain (23.2% in FY2015 vs. 24.4% in 2019).

**Table 2:** Distribution of indications for opioid initiation in fiscal year 2015 and calendar year 2019.

Indication	April 1, 2015 to March 31, 2016 N = 653,885	January 1 to December 31, 2019 N = 571,652	Standardized difference
**Dental**	151,873 (23.2%)	139,448 (24.4%)	0.03
Dentist prescribed	144,117 (22.0%)	134,328 (23.5%)	0.03
Physician prescribed	7,756 (1.2%)	5,120 (0.9%)	0.03
**Cancer and palliative**	42,744 (6.5%)	43,367 (7.6%)	0.04
Palliative care	7,891 (1.2%)	11,382 (2.0%)	0.06
Cancer	34,853 (5.3%)	31,985 (5.6%)	0.01
**Surgery**	113,603 (17.4%)	128,535 (22.5%)	0.13
Hernia repair	10,900 (1.7%)	11,651 (2.0%)	0.03
Knee, hip, and shoulder surgery	18,320 (2.8%)	24,735 (4.3%)	0.08
Common excision	27,370 (4.2%)	30,681 (5.4%)	0.06
Other surgery	50,974 (7.8%)	53,662 (9.4%)	0.06
Caesarean section	6,039 (0.9%)	7,806 (1.4%)	0.04
**Trauma**	73,069 (11.2%)	53,661 (9.4%)	0.06
Fracture and major trauma	22,581 (3.5%)	19,079 (3.3%)	0.01
Dislocations, sprains, and strains	26,341 (4.0%)	18,780 (3.3%)	0.04
Burns, wounds, and superficial trauma	14,722 (2.3%)	9,699 (1.7%)	0.04
Other trauma	9,425 (1.4%)	6,103 (1.1%)	0.03
**Musculoskeletal pain**	78,155 (12.0%)	55,239 (9.7%)	0.07
Back	31,693 (4.8%)	21,841 (3.8%)	0.05
Joint and muscle	46,462 (7.1%)	33,398 (5.8%)	0.05
**Other types of pain**	115,973 (17.7%)	85,943 (15.0%)	0.07
Nephrolithiasis/cholecystitis	15,052 (2.3%)	12,714 (2.2%)	0.01
Headache and migraine	5,335 (0.8%)	3,318 (0.6%)	0.03
Infection	18,976 (2.9%)	11,588 (2.0%)	0.06
Eyes, ears, nose, and throat	14,860 (2.3%)	10,454 (1.8%)	0.03
Abdominal/pelvic pain	38,916 (6.0%)	28,859 (5.0%)	0.04
Chest pain	10,628 (1.6%)	7,581 (1.3%)	0.02
Nonsurgical deliveries	5,848 (0.9%)	6,347 (1.1%)	0.02
Other pain	6,358 (1.0%)	5,082 (0.9%)	0.01
**Unknown**	78,468 (12.0%)	65,459 (11.5%)	0.02

Across all indications, there was a small reduction in the prevalence of new users receiving an initial daily dose above 50MME (23.9% in FY2015 vs. 20.1% in 2019). We observed significant reductions in the prevalence of high dose opioid prescribing across all indication clusters, with the exception of dental pain, which was driven by a lack of change in dentist-prescribed opioids (13.6% vs. 12.1% of prescriptions with daily dose >50MME in FY2015 vs. 2019; Std Diff 0.04; Table 3). The post-surgical pain indication cluster had the highest prevalence of opioid initiation above 50MME in both annual cohorts, but this declined from 40.5% to 34.5% between FY2015 and 2019. However, this varied considerably by surgery type, with the largest changes observed among surgeries for hernia repair, common excisions, Caesarian sections, and other non-orthopedic surgeries. We observed no reduction in the prevalence of high dose opioid initiation among people undergoing knee, hip and shoulder surgery, the indication category with the greatest prevalence of high-dose opioid initiation, with nearly two-thirds of initial opioid prescriptions for this indication having a daily dose above 50MME (64.7% vs. 62.1% in FY2015 vs. 2019; Std Diff 0.05).

**Table 3:** Prevalence of high initial dose and duration among new opioid recipients, by indication. Fiscal year 2015 and calendar year 2019.

Indication	No. (%) with daily dose >50 MME			No. (%) with days’ supplied >7		
	FY2015	2019	Standardized difference	FY2015	2019	Standardized difference
**Overall**	**156,461 (23.9%)**	**115,042 (20.1%)**	**0.09**	**113,517 (17.4%)**	**84,378 (14.8%)**	**0.07**
**Dental**	**21,158 (13.9%)**	**16,897 (12.1%)**	**0.05**	**5,721 (3.8%)**	**5,192 (3.7%)**	**0**
Dentist prescribed	19,626 (13.6%)	16,271 (12.1%)	0.04	5,037 (3.5%)	4,719 (3.5%)	0
Physician prescribed	1,532 (19.8%)	626 (12.2%)	0.21	684 (8.8%)	473 (9.2%)	0.01
**Cancer and palliative**	**12,979 (30.4%)**	**9,578 (22.1%)**	**0.19**	**9,315 (21.8%)**	**8,326 (19.2%)**	**0.06**
Palliative care	1,819 (23.1%)	1,960 (17.2%)	0.15	2,777 (35.2%)	3,322 (29.2%)	0.13
Cancer	11,160 (32.0%)	7,618 (23.8%)	0.18	6,538 (18.8%)	5,004 (15.6%)	0.08
**Surgery**	**45,993 (40.5%)**	**44,322 (34.5%)**	**0.12**	**12,360 (10.9%)**	**10,732 (8.3%)**	**0.09**
Hernia repair	3,735 (34.3%)	2,895 (24.8%)	0.21	591 (5.4%)	259 (2.2%)	0.17
Knee, hip, and shoulder surgery	11,855 (64.7%)	15,355 (62.1%)	0.05	4,182 (22.8%)	4,875 (19.7%)	0.08
Common excision	8,476 (31.0%)	7,157 (23.3%)	0.17	2,555 (9.3%)	1,887 (6.2%)	0.12
Other surgery	18,892 (37.1%)	16,206 (30.2%)	0.15	4,808 (9.4%)	3,435 (6.4%)	0.11
Caesarean section	3,035 (50.3%)	2,709 (34.7%)	0.32	224 (3.7%)	276 (3.5%)	0.01
**Trauma**	**18,263 (25.0%)**	**10,498 (19.6%)**	**0.13**	**15,475 (21.2%)**	**11,173 (20.8%)**	**0.01**
Fracture and major trauma	7,539 (33.4%)	4,852 (25.4%)	0.18	3,235 (14.3%)	2,363 (12.4%)	0.06
Dislocations, sprains, and strains	5,287 (20.1%)	3,214 (17.1%)	0.08	8,775 (33.3%)	6,412 (34.1%)	0.02
Burns, wounds, and superficial trauma	3,116 (21.2%)	1,439 (14.8%)	0.17	2,086 (14.2%)	1,412 (14.6%)	0.01
Other trauma	2,321 (24.6%)	993 (16.3%)	0.21	1,379 (14.6%)	986 (16.2%)	0.04
**Musculoskeletal pain**	**17,479 (22.4%)**	**9,427 (17.1%)**	**0.13**	**26,768 (34.2%)**	**18,311 (33.1%)**	**0.02**
Back	6,532 (20.6%)	3,043 (13.9%)	0.18	9,253 (29.2%)	6,257 (28.6%)	0.01
Joint and muscle	10,947 (23.6%)	6,384 (19.1%)	0.11	17,515 (37.7%)	12,054 (36.1%)	0.03
**Other types of pain**	**27,209 (23.5%)**	**14,930 (17.4%)**	**0.15**	**18,746 (16.2%)**	**12,233 (14.2%)**	**0.05**
Nephrolithiasis/cholecystitis	5,853 (38.9%)	3,162 (24.9%)	0.30	953 (6.3%)	648 (5.1%)	0.05
Headache and migraine	593 (11.1%)	233 (7.0%)	0.14	1,497 (28.1%)	999 (30.1%)	0.05
Infection	3,091 (16.3%)	1,414 (12.2%)	0.12	4,202 (22.1%)	2,514 (21.7%)	0.01
Eyes, ears, nose, and throat	1,848 (12.4%)	1,103 (10.6%)	0.06	2,819 (19.0%)	1,839 (17.6%)	0.04
Abdominal/pelvic pain	10,442 (26.8%)	5,655 (19.6%)	0.17	5,047 (13.0%)	3,235 (11.2%)	0.05
Chest pain	2,127 (20.0%)	1,143 (15.1%)	0.13	2,645 (24.9%)	1,870 (24.7%)	0.01
Nonsurgical deliveries	1,565 (26.8%)	1,178 (18.6%)	0.20	290 (5.0%)	191 (3.0%)	0.10
Other pain	1,690 (26.6%)	1,042 (20.5%)	0.14	1,293 (20.3%)	937 (18.4%)	0.05
**Unknown**	**13,380 (17.1%)**	**9,390 (14.3%)**	**0.07**	**25,132 (32.0%)**	**18,411 (28.1%)**	**0.09**

In contrast, there was little change in the prevalence of initial prescription durations longer than 7 days (17.4% in FY2015 vs. 14.8% in 2019; Std Diff 0.07). Despite already being relatively low, we observed reductions in longer duration prescriptions following surgeries for hernia repair, common excisions, and other non-orthopedic surgeries, but no meaningful differences among people starting opioids following a Caesarian section or knee, hip or shoulder surgery (Table 3). There were also reductions in long-duration initial prescriptions among palliative care patients (35.2% vs. 29.2%; Std Diff 0.13). Clinical indications with a consistently higher than average prevalence of long initial prescription durations in both annual cohorts (FY2015 vs. 2019) were palliative care (35.2% vs. 29.2%), back pain (29.2% vs. 28.6%), joint and muscle pain (37.7% vs. 36.1%), dislocations, sprains and strains (33.3% vs. 34.1%), and headache and migraine (28.1% vs. 30.1%).

## Discussion

In this population-based study of all Ontarians initiating opioid treatment for pain in two annual cohorts, we found a 12.6% reduction in the number of people initiating opioids, and small reductions in the initial daily opioid dose prescribed following the release of guidelines for opioid use in chronic non-cancer pain. Despite these reductions, the initiation of opioid therapy at doses exceeding thresholds currently recommended in guidelines continues to be relatively high, with 1 in 5 people receiving their first outpatient prescription for an opioid with a daily dose above 50 MME in 2019. However, this varies by indication, with only 12% of prescriptions for dental pain exceeding this threshold, while prescriptions for orthopedic joint replacement pain commonly exceed the threshold (62.1%). In contrast, there have been much smaller changes in the duration of opioid prescriptions, with approximately 1 in 7 patients newly treated with opioids receiving more than a 7-day duration in 2019. There is much more variation in the prevalence of longer duration prescriptions across indications, with this practice being more rare among people being treated for conditions that are more likely associated with short-term analgesia needs (e.g. post-surgical pain, dental pain, and postpartum pain).

Recently published studies have indicated that incident opioid exposure, as well as high dose, and long-duration opioid prescribing have been declining across North America since 2012 [14,21–23]. In one study evaluating the impact of the U.S. guidelines on patterns of opioid initiation, rates of new opioid use and initial doses above 50 MME lowered in years following the publication of the guidelines, although the authors noted that these trends began prior to the release of the guidelines and therefore may not have been influenced directly by the guidelines themselves [14]. Interestingly, the prevalence of both long duration and high dose opioid prescribing were highly consistent with those in our study. Specifically, 16% of new opioid prescriptions (2012–2017) exceeded 7 days supply and 22% had an initial dose above 50 MME in the U.S. [14] compared to 15% and 20%, respectively in 2019 in our study. This demonstrates a high degree of consistency in opioid-related prescribing practice between these jurisdictions that is not well aligned with recommendations in clinical guidelines. Although there is evidence of practice changes over time, they appear to be slow, suggesting a need for improved integration of evidence-informed recommendations into clinical practice.

In contrast to studies in the U.S., our study found no significant reduction in longer duration initial opioid prescriptions after the publication of Canadian guidelines, which may reflect differences in the recommendations between national guidelines. In particular, the U.S. guideline includes recommendations for clinicians when using opioids for acute pain, suggesting that durations longer than 7 days are rarely needed [6]. Although an Ontario quality standard for acute pain made a similar recommendation, the Canadian national guidelines for chronic non-cancer pain made no recommendations related to duration of initial prescriptions [7,8]. Given that recent research has demonstrated an association between duration of initial prescription and risks of long-term opioid use and harm [24], more efforts to reduce initial prescription durations may be needed. Particular focus should be paid to indications for which we found a high prevalence of long-duration prescriptions despite weak justification for opioid therapy (e.g. headache/migraine) [25] and where pain is likely acute (e.g. dislocation, sprain, or strain).

In 2019, almost half of all people initiating opioid therapy were being treated for dental or post-surgical pain. Although these represent two very different patient populations, this finding identifies two clinical areas where attempts to promote appropriate opioid initiation practices could be particularly impactful. Importantly, among people prescribed opioids for dental pain, there was no change in the prevalence of high daily doses or longer durations at initiation, despite the publication of the Canadian guidelines (2017), the Ontario quality standard for acute pain (2018), and a dental opioid prescribing guideline (2015) [26]. Although long-duration opioid prescribing was relatively rare for this indication (4% in 2019), the high prevalence of use (24% of all new opioid starts), and considerable degree of high dose prescribing at initiation (12% >50 MME) warrants attention by dental professionals and regulatory bodies. In contrast, despite slight reductions in the initial doses and durations of opioids prescribed post-surgically, over one-third of these prescriptions had daily doses above 50 MME and 1 in 12 had a duration longer than 7 days. This aligns with literature that has demonstrated a reliance on post-surgical opioid prescribing in North America, despite evidence suggesting its contribution to long-term opioid use and opioid-related harm [27,28]. Interventions focused on individualizing discharge prescriptions following surgery, and undertaking shared decision-making with patients to set expectations for pain management post-surgery are needed to reduce potentially inappropriate opioid prescribing in this setting [28,29].

A core strength of this study is that it leverages population-based administrative data on all prescription opioids dispensed in Ontario in two annual cohorts, allowing us to compare how clinical practice has shifted following the release of new national guidelines. However, several limitations merit mention. First, our prescription monitoring program database does not capture the indication for the opioid dispensed; we relied upon diagnosis and procedure codes from recent healthcare encounters to infer indication. However, this approach is consistent with methods reported elsewhere [15] and the proximity of the healthcare encounters to the initial opioid dispenses increases our confidence that the inferred indications are valid. It is also possible that some people had multiple indications at time of opioid initiation, and our hierarchy precluded us from classifying each of these indications. However, the hierarchy was developed to preferentially assign people according to the most appropriate indication for opioid use. Second, we only have opioid dispensing data from July 2012 onwards, and therefore it is possible that people included in our study had more remote opioid use. However, in both cohorts, we looked back at least 33 months, and therefore any previously acquired tolerance would have been lost. Third, some people likely initiated their opioids as an inpatient, and therefore their first outpatient prescription would not represent their first exposure to opioids in their course of treatment. While this could lead to some dose escalation prior to discharge from hospital, it is unlikely that length of stays in hospital would be sufficient to justify a dose escalation beyond 50 MME. Fourth, we report changes in opioid prescribing patterns over time by comparing the prevalence of opioid initiation outside of published recommendations in two annual cohorts. Therefore, we are unable to evaluate the specific impact of the guideline and quality standards themselves because it is possible that some of the observed changes reflect broad shifts in clinical practice in Ontario, rather than being directly attributable to these publications. Therefore, while this study can demonstrate the degree to which some aspects of clinical practice have shifted after the publication of these guidelines, we cannot directly attribute these changes to the guideline and quality standards themselves and cannot preclude the possibility that other elements of clinical practice were impacted by their publication. Finally, we are unable to capture pain severity in our data, and therefore are unable to determine whether reductions in the overall incidence of opioid initiation, or changes in initial prescription characteristics were appropriate in all cases. Although there is evidence internationally that a lower reliance on opioids does not broadly result in patient harm [28,29], opioids play an important role in managing severe pain, and ongoing efforts are needed to ensure that clinical and policy opioid responses are both evidence-based and tailored to individual patient needs.

## Conclusion

Although the number of people initiating prescription opioids declined across Ontario between 2015 and 2019, there have been few changes in opioid prescribing practices at time of initiation. With 1 in 5 people newly initiating opioids in 2019 being prescribed a daily dose that exceeds 50 MME, and 1 in 7 receiving more than a week supply at initiation, substantial changes in clinical practice would be needed in order to align with recommendations from national clinical guidelines. Given the variation observed between pain indications, messaging should be tailored within clinical specialties, with focused efforts in the areas of dental and post-surgical pain likely being most impactful.

## Supporting information

S1 Dataset (DOCX)

S1 File (ZIP)
