# Supplementary material for: Changing patterns of opioid initiation for pain management in Ontario, Canada: A population-based cross-sectional study
Source: PLoS One. 2022 Dec 8;17(12):e0278508. doi: 10.1371/journal.pone.0278508 (PMC9731435; doi:10.1371/journal.pone.0278508)
Supplement: S1 Dataset — (DOCX) [file pone.0278508.s001.docx]

| Project Identification | |
| --- | --- |
| **ICES Project Title:** | Clinical Indications Associated with Opioid Initiation: A Follow-Up Study |
| **Project Objectives:** | *Insert Project Objectives as listed in the approved ICES Project PIA* |
|  | To examine the clinical indications associated with opioid initiation for pain management among Ontarians starting opioid therapy following the release of the 2017 Canadian Guideline for Opioids for Chronic Non-Cancer Pain |

| Project Cohort | | |
| --- | --- | --- |
| **Study Design** | Cohort study  Matched cohort study  Case-control study  Cross-sectional study  Other (specify): | |
| **Cohort 1: FY2015 Cohort** | | |
| **Study Period** | Study Period: April 1, 2015 – March 31, 2016 | |
| **Cohort** | - Note: this cohort was created for a previously published study (available: <https://www.ncbi.nlm.nih.gov/pmc/articles/PMC6085129/>). - Using this cohort, apply the following additional exclusion: Remove people who **only** had opioid prescription where dclass in (‘EXCL’, ‘COUGH’) or dclass_broad in (‘RUNS’, ‘MAID’) on their index date | |
| **Cohort 2: CY2019 Cohort** *(Note: this cohort created using same methods as FY2015 cohort above as outlined below)* | | |
| **Study Period** | January 1, 2019 to December 31, 2019 | |
| **Cohort** | New users of opioids for pain   - Opioids for pain defined in NMS Master Drug List as NMS_GROUP=’OPIOID’, OMT=’N’, dclass^=(’EXCL’, ‘COUGH’), and dclass_broad^=(‘RUNS’,‘MAID’). - New use is defined as having no opioid prescription since April 1, 2016 (include the index date when looking back for opioid agonist therapy, exclude when looking back for all other types of opioids) | |
| **Index Prescription** | First prescription in study period   - Note: If there is more than one prescription on index, keep all | |
| **Index Date** | Date of first prescription in the study period | |
| **Exclusions** *(in order)* | *Step* | Description |
|  | 1 | *Missing IKN (unique patient identifier), invalid IKN, or cardholder_ID ne ‘ON’* |
|  | 2 | *Prior opioid prescription for pain, cough, or diarrhea (NMS_GROUP=’OPIOID’ and OMT=’N’) before index date (look back to Apr. 1, 2016)* |
|  | 3 | *Prior prescription for opioid agonist therapy (NMS_GROUP=’OPIOID’ and OMT=’Y’) before or on the index date (look back to Apr. 1, 2016)* |
|  | 4 | *Opioid toxicity incident in the 2 years prior to (not including) the index date (see diagnosis codes and macro parameters in* [*Appendix A*](#_Appendix_A:_Codes)*)* |
|  | 5 | *Death date prior to index date (. < DTHDATE < INDEXDATE)* |
|  | 6 | *Invalid age in RPDB (negative age, age > 105, missing age when linked to RPDB)* |
|  | 7 | *Non-Ontario resident on the index date (substr(prcddablk,1,2) ne ‘35’)* |

| Part 1: Ascertainment of Clinical Indication for Opioid Initiation | | |
| --- | --- | --- |
| **Licence ID** | **Please provide procedure frequency of licence_id_ref on the index rx before proceeding.**  **Note: if licence_id_ref = ‘2’, then change to ‘02’**  **Note: if licence_id_ref in (‘1’ ‘A1’ ‘MD’), then change to ‘01’** |  |
| **Prescription Setting** | For each person starting opioid therapy, classify prescription indication/setting using a hierarchal approach as follows:   1. Dental prescribing    1. If licence_id_ref = ‘02’ on index Rx, then SETTING = “DENTIST”    2. EXCEPT if person had both ‘01’ and ‘02’ at index. In this case, classify as ‘01’. 2. Palliative care    1. If any of the following criteria are met within 1 year prior to and including the index date then SETTING = “PALLIATIVE”       1. Any CIHI-DAD hospitalization with patient service (PATSERV) = 58. (source=inpatient, acute care hospital=T), **OR**       2. Any OHIP billing with one of the following billing codes: OHIP FEECODE A945, B998, C945, C882, C982, K023, W872, W882, W972 or W982.          - Spec=all, **OR**       3. Any ALR visit (use Visit_Date variable) with any one of the following:          - Visit_Program_Code=PA (ALR Clinic Visit dataset)          - Intent_of_systemic_treatment=P (ALR Systemic dataset)          - Intent_of_radiation=P (ALR Radiation dataset) 3. Cancer    1. Look for ANY OF THE FOLLOWING as evidence of recent cancer **diagnosis** in the 1 year prior to and including the index date:       1. Use OCR.incident_cases2019 – any individual identified in this database with “dxdate” in the past 1 year    2. Look for ANY OF THE FOLLOWING as evidence of cancer **treatment** within the past 1 year prior to and including the index date       1. Use the ALR clinic visit dataset to check for BOTH of          - Visit_Date within past 1 year          - Visit_Program_Code = RAD, SUR, SC, or SYS       2. Use OHIP Feecodes and DxCodes to check for BOTH of          - Chemotherapy or radiation: Feecodes G281 G339 G345 G359 G381 G382, X310 X311 X312 X313          - NO diagnosis of RA/Crohn’s (DxCodes 714, 555) on the same visit (same record) as chemo/radiation          - Spec=all          - Source=nonlab          - Servdate in the past 1 year    3. If any evidence of cancer diagnosis or treatment in the prior 1 year, then SETTING = “CANCER” 4. Other Indications - define Setting based on following:    1. Flag recent (index or five days prior) healthcare visit       1. Any ED visit on index date or five days prior (using NACRS REGDATE)          - Include scheduled visits       2. Any Same Day Surgery (SDS) visit on index date or five days prior (using SDS admdate)          - Exclude records where incode1= ‘CANCELLED’       3. Any hospital discharge on index date or five days prior (using CIHI DAD ddate)          - Include acute and non-acute institutions       4. Any physician visit on index date or five days prior (using OHIP servdate)          - %getohip parameters:            1. Spec=phys (physicians only)            2. Limit to location= (O,L,P,H)   Note:OHIP claims with location=U (Undefined) will be checked at the last step for the N(%) in the ‘UNKNOWN’ setting group who may have had a physician visit   - - - - 1. Source=nonlab   1. Use the following method to assign these people a SETTING from one of 6 (ED, SDS, HOSP, PHYSICIAN, TBD, UNKNOWN)      1. If ONLY had ED, then SETTING = “ED”      2. If ONLY had Same Day Surgery, then SETTING = “SDS”      3. If ONLY had hospital discharge, then SETTING = “HOSP”      4. If ONLY had physician visit, then SETTING = “PHYSICIAN”      5. If physician visit and ONE OF hospital discharge/ED/SDS, then         - Use presc_i on prescription (prescriber ID on prescription), convert to physnum (OHIP physician number)           1. Limit these to licence_id_ref in (’01’)           2. Some presc_i convert to two physnum, keep all physnums.         - Note: some individuals had more than one office visit on index or 5 days prior. Keep all records.         - If physician prescribing opioid is *same* as physician in office visit, then SETTING = “PHYSICIAN”           1. Given that some patients have more than one physnum associated with index Rx and some patients have more than one physnum associated with office visits on index or five days prior, if for each IKN there is ANY match of physnums between index rx physnums and office visit physnums, then setting= ‘PHYSICIAN’         - Otherwise, then SETTING = “ED”, “HOSP”, “SDS” (whichever is relevant)      6. If individual had physician office visit and MORE THAN ONE OF hospital discharge/ED/SDS, then         - Use Presc_i on prescription, convert to physnum         - If physician prescribing opioid is *same* as physician in office visit, then SETTING = “PHYSICIAN”           1. Note: some presc_i link to two physnums. If in those cases, if either of the physnums that linked to one presc_i match the physnum on OHIP, then setting=PHYSICIAN.         - Otherwise, SETTING = “TBD”      7. If individual had MORE THAN ONE OF hospital discharge/ED/SDS, BUT NOT physician office visit, then         - SETTING = “TBD”.      8. If individual had NONE of the 4, then SETTING = “UNKNOWN”         - Of those categorized as unknown, check how many had a physician visit on index or 5 days prior with location=undefined (physnum match not necessary)         - Of those categorized as unknown, check how many had a discharge from inpatient rehabilitation on index or 5 days prior (use NRS DISCHARGE data, reference to discharge date)   **There should be 9 prescription settings in the end (DENTIST, PALLIATIVE, CANCER, ED, SDS, HOSP, PHYSICIAN, TBD, UNKNOWN).** |  |
| **Indication** | Indication is dependent upon SETTING as described below:   1. If SETTING = DENTIST, then INDICATION = “DENTAL” 2. If SETTING = CANCER, then INDICATION = “CANCER” 3. If SETTING = PALLIATIVE, then INDICATION = “PALLIATIVE” 4. If SETTING = SDS then, provide frequency distribution **ALL INCODE variables** (only 5 character length).    1. Note – if more than one discharge record within index or 5 days prior for each IKN, keep all records because we don’t know which one may have been responsible for opioid Rx.    2. Note – exclude INCODES where associated INATSTAT = A (Abandoned)    3. Attempt to categorize procedures as:       1. **Surgical:** Any Therapeutic Intervention CCI code (first character=1) with 4th and 5th digit equal to 50 or greater (eg. 1. ^^.50.^^) and 4^th^ and 5^th^ character not equal to 52 (drainage)       2. **Obstetric delivery procedures:** Any Obstetrical and Fetal Intervention CCI code (first character=5) and 2^nd^ and 3^rd^ characters NOT “AB” (i.e., exclude antepartum diagnostic interventions) and 4^th^ and 5^th^ character not equal to 73 (drainage)       3. **Dental procedures** (1FF or 1FE or 1FD)    4. If any INCODE is within above surgical, obstetric delivery, or dental procedure, then apply hierarchical classification of INDICATION in this order:       1. “DENTAL PROC” (to distinguish from dental indication from dentists based on licence_id_ref)       2. “DELIVERY PROC”       3. “SURGERY”    5. Otherwise, create a separate dataset with the frequency distribution of DX10CODE1 codes (only 3 character length) from DAD, SDS, ED records and include a column with code DESCRIPTION       1. Note: If more than one discharge record in the 5 day look-back, then keep most recent record that is not missing DX10CODE1 (reason: scale up for opioid prescriptions). If more than one record on the same day that is the most recent, keep all on that day (most pertinent for ED).       2. Note: Some records may be missing DX10CODE1 –classify these into the unknown indication group    6. The INDICATIONS will be set based on clusters of codes (determined by applying indications for codes already categorized in the last project, and grouping any new codes manually based on clinical expertise) 5. If SETTING = HOSP, then follow the same procedure as SDS 6. If SETTING = ED, then follow the same methods as SDS and HOSP   **Provide frequency of INCODES and the assignment of dental procedure, delivery procedure, surgical procedure.**   1. If SETTING = PHYSICIAN, then create a separate dataset with the frequency distribution of DXCODE codes from OHIPDX dataset and include a column with code DESCRIPTION    1. Link to physician records with location=O,L,H,P, U.    2. Data cleaning steps in order performed (D1-D2):       1. D1. Use records where physnum with licence_id_ref= ‘01’ on prescription matches physnum on OHIP record *where possible (i.e.,if IKN had at least 1 matching physnum OHIP record, then keep only records where physnum matched; else if IKN had only non-matching physnums, then keep all non-matching OHIP physnum records)*          - Note: When joining presc_i to physnums on Rx to physnum on OHIP records, OHIP records will be duplicated for two reasons:            1. 1) Some presc_i link to two physnums. If either of those physnums link to the OHIP record, then keep the matching physnums. If none of those prescriber physnums match to any OHIP record, only keep one record to avoid double counting DX codes (doesn’t matter which one).            2. 2) Some IKNs have more than one presc_i on index. If any of the prescriber physnums match the OHIP record, then keep those records. If none of the prescriber physnums match any of the OHIP records, then just keep one of the prescriber physnums to avoid double counting (doesn’t matter which one)       2. D2. For each IKN, keep record(s) on the servdate closest to index date          - If only DXCODE= ‘999’ then indication= ‘UNKNOWN’ and major group =’UNKNOWN’    3. Output a separate dataset with the DXCODE frequencies and associated descriptions for those in the SETTING=PHYSICIAN together with those in the SETTING=UNKNOWN with an undefined location OHIP record in 5 days prior.    4. The INDICATIONS will be set based on clusters of codes (determined by applying indications for codes already categorized in the last project, and grouping any new codes manually based on clinical expertise) 2. If SETTING = UNKNOWN, then do:    1. If physician visit with location=undefined, then output a separate dataset with the frequency distribution of DXCODES along with the DXCODES from setting=PHYSICIAN.       1. Follow same data cleaning steps as SETTING=PHYSICIAN       2. Output a separate dataset with the DXCODE frequencies and associated descriptions for those in the SETTING=PHYSICIAN together with those in the SETTING=UNKNOWN with an undefined location OHIP record in 5 days prior.    2. Else if rehab discharge record then indication= “REHAB”. Note that this will be grouped into the “Other” indication    3. Else indication=UNKNOWN 3. If SETTING = TBD, then do:    1. Check whether any with TBD can be classified into indication=Dental procedures, Delivery related procedures, surgical procedures and classify accordingly (hierarchical)   If they aren’t classified into the dental, delivery, or surgical procedures, then follow the same methods as above for DX10CODES and output along with the setting=SDS, DAD, ED groups  In the original project, Sachin Pasricha, Mina Tadrous, and Tara Gomes created major groups and indications based on the procedure, ICD10, and OHIPDX codes, along with a hierarchical classification scheme in case people have more than one potential indication or major group. These lists were used to create mutually exclusive major groups indications for each IKN. We will use these lists to classify indications for the same diagnosis codes.  For any new diagnosis codes that did not come up in the last project, Tara and Mina will manually review and assign them to major groups and indications based on the procedure, ICD10, and OHIPDX codes.  After all of the indications have been assigned, make the following change in the code to align with a change made in the original project:  If indication=’CSECTION’ then majorgroup=’SURGERY’  If indication=’OTHERDELIVERIES’ then majorgroup = ‘OTHERPAINSOURCES’  If indication =’DENTAL PROC’ then majorgroup=’DENTAL’ and indication=’Physician Prescribed’  If indication = ‘REHAB’ then majorgroup=’Other types of pain” and indication=’Other pain’  After making the change, create an Excel file with a ‘master list’ of procedure codes, ICD10 codes, and OHIPDX codes (each on separate sheets) with their associated major groups and indications. We will save this file for future reference.  Prescription indication and indication cluster (major group) will be classified using the following categories:  **Prescription Indication Cluster and Indication**   - Dental   - Dentist prescribed   - Physician prescribed - Surgery   - Common excision   - Knee, hip, and shoulder surgery   - Hernia repair   - Caesarean section   - Other surgery - Musculoskeletal pain   - Joint and muscle   - Back - Trauma   - Dislocations, sprains, and strains   - Fracture and major trauma   - Burns, wounds, and superficial trauma   - Other trauma - Cancer and palliative   - Cancer   - Palliative - Other types of pain   - Abdominal/pelvic pain   - Infection   - Nephrolithiasis/cholecystitis   - Eyes, ears, nose, and throat   - Chest pain   - Nonsurgical deliveries   - Headache and migraine   - Other pain - Unknown   For the unknown indication, report the source of data (not mutually exclusive):   - No health care record identified (n,%) - OHIP claim (location=O, P, L, H, U) (n,%) - ED (n,%) - DAD (n,%) - SDS (n,%) - NRS (n,%) |  |

| Part 2: Comparison of Person- and Prescription-Level Characteristics Between FY2015 and CY2019 | | |
| --- | --- | --- |
| **Step 1** | Require folder and dataset access to the previous project to get FY2015 cohort. |  |
| **Step 2** | Between FY2015 and CY2019, report:   1. Baseline characteristics of people initiating opioid therapy overall, add standardized difference    - Age group      - Median (IQR)      - 0-17, 18-24, 25-44, 45-64, 65+ (n,%)    - Sex (n,%)    - Neighbourhood income quintile (n,%)    - Urban vs. rural location of residence (n,%)    - Type of opioid on index date (n,%). Report the opioid formulation (long-acting ONLY vs. immediate release ONLY vs. both)    - Daily dose of initial prescription (in MME) (n,%)      - Median (IQR)      - Group into <20, 20-49, 50-89, 90-199, >=200, unknown      - Prescriptions with daily dose > 50 MME    - Days supplied of initial prescription (NMS dayssupl variable) (n,%)      - Median (IQR)      - Group into 1, 2-3, 4-6, 7, 8-13, 14, 15-29, 30, 31+ days      - Prescriptions with dayssupl > 7 2. Frequency and characteristics of initial opioid prescriptions for pain, overall and by major and minor clinical indication, add standardized difference    - Number and percent of people    - Daily dose > 50 MME (n,%)    - Days supplied > 7 (n,%) |  |

| Analysis Plan and Dummy Tables |
| --- |
| **Descriptive Tables** |
| **Table 1. Baseline characteristics of new opioid recipients in fiscal year 2015 and calendar year 2019.** |
| **Table 2. Distribution of indications for opioid initiation in fiscal year 2015 and calendar year 2019.** |
| **Table 3. Prevalence of high initial dose and duration among new opioid recipients, by indication. Fiscal year 2015 and calendar year 2019** |

# Appendix A: Codes for Covariates

| **Covariate** | **Data Source** | **Codes** | **Macro Parameters** |
| --- | --- | --- | --- |
| Opioid toxicity | DAD, NACRS | **ICD-10:**  T400-T404 or T406 | **DAD:** source = inpatient,  refdate = ddate, dxtype = admitdx, inclsuspect = F, acute = T  **NACRS:** source = ed, inclscheduled = F, inclsuspect = F, dedup = T, inclfrom_typee = T,  Inclto_typeip = T, inclnotseen = T, dxtype = alldx |
